# Ascorbic acid-induced warfarin resistance after breast cancer surgery: a case report and literature review

**DOI:** 10.3389/fphar.2024.1390996

**Published:** 2024-04-26

**Authors:** Pingfa Gao, Yang Shen, Ping Wu, Wenjie Lv

**Affiliations:** ^1^ Department of Thyroid and Breast Surgery, Chongming Hospital Affiliated to Shanghai University of Medicine and Health Sciences, Shanghai, China; ^2^ Shanghai University of Medicine and Health Sciences, Shanghai, China; ^3^ Department of Breast Surgery, Xinhua Hospital Affiliated to Shanghai Jiao Tong University School of Medicine, Shanghai, China

**Keywords:** ascorbic acid, warfarin resistance, pharmacokinetics, pharmacodynamics, case reports

## Abstract

Warfarin is an anticoagulant that requires INR-based dosage adjustment. Ascorbic acid may impair warfarin effectiveness according to limited literature. We report a rare case of a 63-year-old woman with an aortic valve replacement history who developed warfarin resistance after taking ascorbic acid for anemia following breast cancer surgery. Despite increasing the warfarin dose from 6 mg to 10 mg daily, her INR remained below the therapeutic range. After ruling out other causes of warfarin resistance, we discontinued ascorbic acid and observed a rapid increase in INR to target values. The temporal relationship and the absence of other confounding factors confirmed the causality of ascorbic acid in this case. We recommend that patients concomitantly taking vitamin C and warfarin should monitor their INR values closely and discontinue ascorbic acid as soon as possible if they exhibit signs of warfarin resistance.

## 1 Introduction

Warfarin is a common anticoagulant for the prevention and treatment of thromboembolic diseases, such as artificial valve replacement, atrial fibrillation, venous thrombosis, and pulmonary embolism (Ansell et al., 2004). The optimal dose of warfarin is determined by the International Normalized Ratio (INR), which reflects the coagulation status of the blood. Warfarin interacts with many drugs, but the interaction with ascorbic acid (vitamin C) has been rarely reported. This report details a rare instance of warfarin resistance, a condition characterized by an unusually high tolerance to the anticoagulant, necessitating increased dosages for efficacy, which in this case, was induced by ascorbic acid.

## 2 Clinical case

**FIGURE 1:**
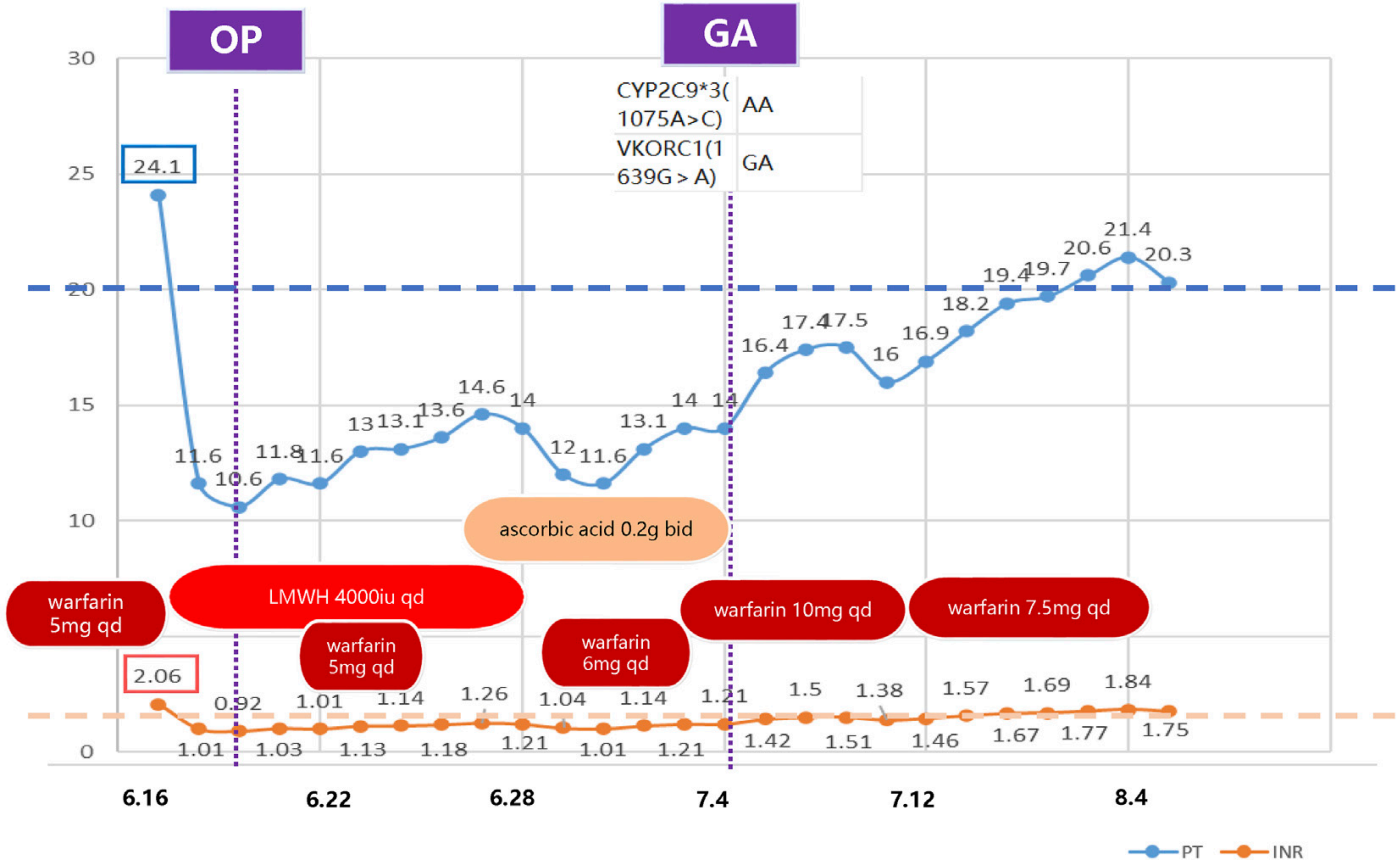
Timeline of clinical medication and laboratory indicators. This figure illustrates the anticoagulant medication and laboratory indicators (PT and INR) during the treatment period. The boxes on the left indicate the baseline values. This demonstrates that the PT and INR failed to reach the baseline level after restarting warfarin postoperatively. The indicators gradually returned to normal after discontinuing ascorbic acid. OP: operation; GA: genetic analysis; LMWH: low molecular weight heparin; qd: quaque die (once a day); bid: bis in die (twice a day); PT: Prothrombin Time; INR: International Normalized Ratio.

We report a rare case of warfarin resistance induced by ascorbic acid in a 63-year-old Chinese woman who underwent right modified radical mastectomy for breast cancer. She had a history of partial gastrectomy (reason unknown) more than 30 years ago and aortic valve replacement 16 years ago, and had been on warfarin anticoagulation since then. Her usual warfarin dose was 7.5 mg qd (once a day), and her INR was around 1.8. She also had thrombocytopenia (preoperative platelet count around 65*10^9^/L) and leukopenia since 2020. She presented with gingival bleeding 1 week before admission and subcutaneous ecchymosis on her left thigh on physical examination. She received interleukin-1 for thrombocytopenia. She was admitted on June 15 for breast cancer surgery and switched from warfarin to heparin sodium bridging (4000iu q12h, subcutaneous) based on the cardiothoracic surgery consultation, and discontinued it 1 day before surgery. She had the surgery on June 20 and resumed heparin sodium (4000iu q12h, subcutaneous) and warfarin anticoagulation (5 mg qd) on June 21, with an INR of 1.03. Heparin sodium was stopped on June 28. Warfarin anticoagulation was temporarily withheld due to incisional bleeding on June 24 (INR 1.13), and warfarin sensitivity gene testing was performed. Warfarin genetic testing primarily involves two genes: VKORC1 and CYP2C9. VKORC1 is the pharmacological target of warfarin, while CYP2C9 is the main enzyme responsible for its metabolism. Genetic variations in these genes can significantly influence an individual’s response to warfarin, thus affecting the necessary dosage and overall efficacy of treatment (Bhatt, 2014). The gene test result showed CYP2C93(1075A>C) AA and VKORC1(1639G>A) GA genotypes, ruling out gene-related warfarin resistance. She was given ascorbic acid (0.2 g bid orally) and ferrous succinate tablets (0.2 g bid orally) to correct anemia due to hemoglobin 69 g/L on June 27. The reason for choosing ascorbic acid is based on the fact that following surgery, the patient exhibited symptoms of iron-deficiency anemia, a condition precipitated by sustained blood loss from the surgical incision. Moreover, it has been reported that, besides its antioxidant property (Teucherl et al., 2004), ascorbic acid can directly improve the sensitivity of the erythropoietin hormone and the uptake and recycling of iron (Lynch and Cook, 1980; Sabatier et al., 2020). She restarted oral warfarin (6 mg qd) on June 29, but her INR did not return to the preoperative level (1.04–1.14) on July 1. The pharmacy department consultation suggested increasing warfarin to 10 mg qd on July 1, but the INR did not rise significantly after the dose adjustment. Upon comprehensive consideration of the patient’s perioperative medication regimen, it is discerned that the short-term preoperative administration of IL-1 does not exert an influence on the anticoagulant efficacy of warfarin. Based on the previous literature, we suspected ascorbic acid-induced warfarin resistance and stopped ascorbic acid on July 4, and the INR increased to 1.21 and then gradually to around 1.5. On July 14, the dose of warfarin sodium was reduced to 7.5 mg, and the INR returned to target range afterwards. The patient was discharged because of her stable INR values (Figure 1). Figure 1 delineates the temporal progression of warfarin and ascorbic acid administration, in conjunction with low molecular weight heparin sodium bridging therapy, and the resultant International Normalized Ratio (INR) and Prothrombin Time (PT) values. Preoperatively, the patient was maintained on a warfarin regimen of 5mg, correlating with an INR of (2.06) and a PT of (24.1 s). Subsequent to warfarin cessation and initiation of low molecular weight heparin bridging, PT and INR values decreased to (10.6 s) and (0.92), respectively, permitting surgical intervention. Postoperative day two witnessed the reintroduction of warfarin at 5 mg daily, which precipitated a gradual elevation in PT. Despite the concomitant bidaily oral administration of ascorbic acid and an escalated warfarin dosage of 10mg, no appreciable augmentation in PT or INR was observed. Cessation of ascorbic acid led to a marked resurgence in both PT and INR values, and a subsequent reduction of warfarin to 7.5 mg daily facilitated a return to baseline preoperative metrics. The patient had good tolerance and no adverse events occurred. INR was stable and within normal range 1 month post-discharge during the follow-up.

## 3 Discussion

**FIGURE 2:**
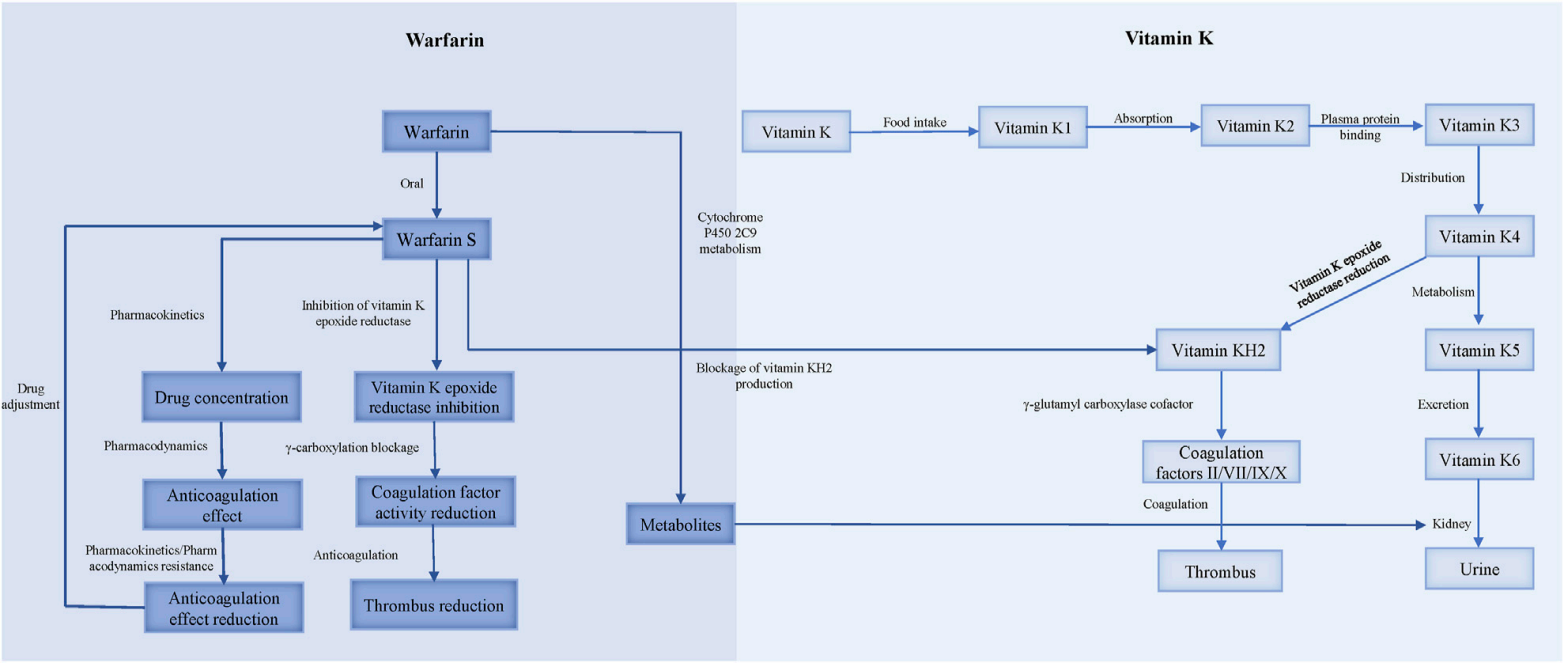
The mechanism of warfarin resistance.

Warfarin resistance, classified into pharmacokinetic and pharmacodynamic types, occurs when the target INR range cannot be achieved with a dose of 10 mg/d or higher (Sinxadi and Blockman, 2008) (Figure 2).

### 3.1 Pharmacokinetic resistance

Pharmacokinetic resistance to warfarin occurs when the organism’s absorption, distribution, metabolism, or excretion processes alter the drug’s effect. Clinically used warfarin is a racemic mixture of R and S isomers, with S-warfarin being 2–5 times more potent as an anticoagulant than R-warfarin. Therefore, INR is more affected by factors that inhibit S-warfarin metabolism (Ansell et al., 2004; Jacobs, 2006). Warfarin is quickly absorbed in the gut, binds to plasma proteins (about 98%), and is metabolized in the liver by CYP2C9. The kidney is the main route of excretion (Hulse, 1996).

The causes of pharmacokinetic resistance can be divided into the following categories: 1) Reduced absorption: This occurs in conditions such as diarrhea, vomiting, or malabsorption syndrome. 2) Increased clearance: This happens when hypoalbuminemia causes the free form of warfarin to increase, leading to faster clearance of warfarin (Diab and Feffer, 1994); or when hyperlipidemia increases the binding rate of lipophilic vitamin K and blood lipids, resulting in a higher total concentration of vitamin K. Patients receiving parenteral nutrition (intravenous infusion of fat-containing and other nutrients) may develop warfarin resistance (MacLaren et al., 1997). Nathisuwan et al.'s meta-analysis showed that smokers need to increase the dose of warfarin, indicating that the clearance rate of warfarin in smokers is enhanced (Nathisuwan et al., 2011). 3) Concomitant medication: Warfarin metabolism involves CYP450 enzymes (CYP2C9 for the S-isomer and CYP1A2, 2C19, 3A4 for the R-isomer), and most drug interactions affecting warfarin inhibit their expression and/or activity. Drugs that induce CYP2C9, such as rifampin, phenobarbital, carbamazepine, and enzalutamide, increase the clearance rate of warfarin. Nafcillin induces CYP3A4 and CYP2C9. Ritonavir induces CYP2C9 and CYP1A2, increasing the clearance rate of the warfarin S-isomer (Holbrook et al., 2005; Mar et al., 2022). St. John’s wort, an herbal antidepressant, reduces warfarin production by inducing CY2C9, 2C19, and 3A4 to increase the clearance rate (Tan and Lee, 2021; Mar et al., 2022). Colesevelam and other bile acid sequestrants inhibit warfarin absorption (Jähnchen et al., 1978; Mar et al., 2022). Diuretics, such as spironolactone, increase coagulation factor levels by reducing plasma volume and cause warfarin resistance (OReilly, 1980) (Table 1). 4) Genetic variation: The gene encoding CYP2C9 affects the clearance rate of warfarin, and genetic variation in this gene can alter warfarin metabolism. Several studies have found that polymorphisms of the genes encoding CYP2C9 and VKORC1 reduce warfarin demand (Gulseth et al., 2009; Zhao et al., 2023). Three main genes guide warfarin dosage: CYP2C9, VKORC1, and CYP4F2. CYP2C9 and VKORC1 explain 35%–50% of the individual difference in warfarin dosage (Johnson et al., 2017). CYP2C92 and CYP2C93 gene mutations reduce CYP2C9 enzyme activity by 30% and 80%, respectively, lowering the clearance rate of warfarin. Patients with the VKORC1 mutant genotype (AA/AG) need a lower warfarin dose and usually do not develop warfarin resistance (Gulseth et al., 2009).

**TABLE 1:** Drug-induced warfarin resistance and its management strategies.

Category	Drug	Mechanism	Clinical management
Antitubercular agents	Rifampicin OReilly (1974), Heimark et al. (1987)	CYP2C9 induction	Closely monitor the INR.
Antibiotics	Nafcillin Kim et al. (2007), King et al. (2018), Mar et al. (2022)	CYP2C9,3A4 induction	Following the discontinuation of the medication for 2–4 weeks, the INR level requires close monitoring for an additional 2–4 weeks
Sedative-hypnotics	Phenobarbital Udall (1975)	CYP2C9 induction	Monitor the INR closely and switch medication if necessary
Anticonvulsants	Carbamazepine Mannheimer et al. (2016)	CYP2C9 induction	Escalate warfarin dosage by 50% and monitor the INR frequently
Antineoplastic agents	Enzalutamide Gibbons et al. (2015), Mar et al. (2022)	CYP2C9 induction	Closely monitor the INR.
Antiretroviral agents	Ritonavir Hull and Montaner (2011), Mar et al. (2022)	CYP2C9,1A2 induction	Closely monitor the INR.
Herbal medicines	St. John’s Wort Tan and Lee (2021), Mar et al. (2022)	CYPC9,2C19,3A4 induction	Closely monitor the INR.
Bile acid-binding resins	Colestyramine Jähnchen et al. (1978), Mar et al. (2022)	Impair the enterohepatic recycling and reduce the uptake	Monitor the INR closely and apply it to counteract warfarin toxicity
Diuretic agents	Spironolactone OReilly (1980)	Elevate the concentration of coagulation factors	Closely monitor the INR.

### 3.2 Pharmacodynamic resistance

Warfarin resistance caused by the reduced anticoagulant effect of pharmacodynamic processes is called pharmacodynamic resistance. The activation of coagulation factors II, VII, IX, X and proteins C, S, Z requires gamma-carboxylation by gamma-glutamyl carboxylase (GGCX), which initiates the blood coagulation cascade. GGCX depends on reduced vitamin K (vitamin KH2) as a cofactor, which is produced by vitamin K epoxide reductase (VKOR) from oxidized vitamin K. CYP4F2 is the enzyme that oxidizes vitamin K, and thus regulates the availability of vitamin KH2 for GGCX. Warfarin inhibits VKOR, and consequently reduces the vitamin KH2 level, impairing the function of GGCX and exerting an anticoagulant effect (Ansell et al., 2004).

Warfarin resistance can occur due to pharmacodynamic factors, such as 1) increased affinity of VKOR for vitamin K, which reduces the availability of the oxidized form of vitamin K which is the substrate for warfarin inhibition (OReilly et al., 1968); 2) decreased affinity of warfarin for VKOR, which reduces the potency of warfarin inhibition. A study on warfarin-resistant rats found that overexpression of a protein called calumenin interferes with the binding of warfarin to VKOR (Daly and Aithal, 2003); 3) expression of coagulation factors that are independent of vitamin K, which bypasses the need for VKOR activity (OReilly, 1970); and 4) enhanced synthesis or activity of coagulation factors, which overwhelms the anticoagulant effect of warfarin. For instance, patients who take ethinyl estradiol for a long time require higher doses of warfarin to achieve adequate anticoagulation (Davies et al., 1976).

### 3.3 Ascorbic acid-warfarin interaction

Pauling (Pauling, 1970) proposed that daily intake of 1 g of ascorbic acid could decrease the incidence of colds by 45% in the majority of individuals, and recommended ascorbic acid therapy for common colds, although some individuals required higher doses (Pauling, 1970; Pauling, 1976). This hypothesis stimulated the extensive use of vitamin C and the identification of its interactions with other drugs.

Sigell and Flessa (1970) were the first to document the interaction between warfarin and ascorbic acid in 1970. The following year, Rosenthal (Rosenthal, 1971) described a 52-year-old female patient with pulmonary embolism who was treated with warfarin. Her prothrombin time (PT) was 23 s at discharge, compared to a normal value of 12 s, with a daily dose of 7.5 mg of warfarin. However, her PT decreased to 14 s 4 weeks later, despite increasing the warfarin dose. Upon further investigation, it was found that she had been taking ascorbic acid (dose unspecified) for a cold. Her PT increased to 28 s after discontinuing ascorbic acid for 2 days. In 1972, Smith et al. (Smith et al., 1972) reported a similar case of a 70-year-old woman who consumed about 16 g of ascorbic acid daily. She exhibited abnormal resistance to warfarin therapy during hospitalization, necessitating 25 mg daily to achieve a significant elevation in PT. She was discharged with a maintenance dose of 10 mg daily. These studies all indicated an interaction between ascorbic acid and warfarin.

**FIGURE 3:**
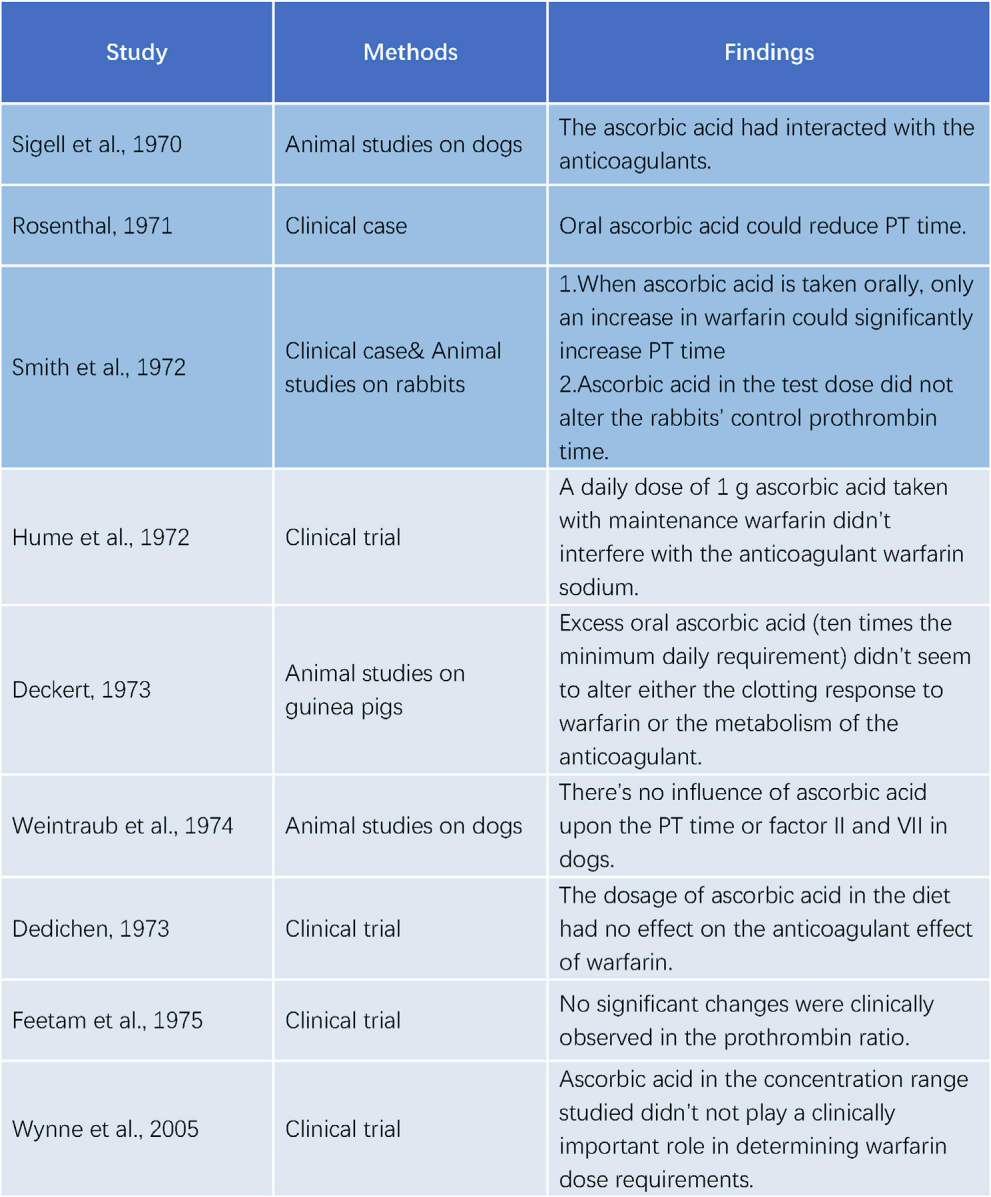
Studies on the interaction between ascorbic acid and warfarin.

Contrary to the initial reports of Sigell and Flessa (1970); Rosenthal (1971); Smith et al. (1972) were unable to replicate the effect of ascorbic acid on warfarin in rabbits. Hume et al. (1972) performed a trial with five patients on long-term warfarin therapy and administered 1 g of ascorbic acid daily. They found no impact of ascorbic acid on the anticoagulant activity of warfarin. Deckert (1973a); Deckert (1973b) also observed no alteration in the coagulation response to warfarin by excessive ascorbic acid in guinea pigs. Weintraub and Griner (1974) measured the concentrations of PT and factor II and VII/X complex in dogs anticoagulated with warfarin and reported no changes after long-term treatment, ruling out a significant drug interaction between warfarin and ascorbic acid. Dedichen (1973) compared the warfarin dose in chronic anticoagulant patients who took 1 g of ascorbic acid daily for 6 months with those who did not and detected no difference, suggesting that the ascorbic acid dose in the diet had no influence on the anticoagulant effect of warfarin. Feetam et al. (1975) studied 19 subjects taking warfarin and showed that, despite a 17.5% average decrease in the total plasma warfarin concentration after taking 3, 5 or 10 g of ascorbic acid daily for 7 days, the prothrombin ratio did not change significantly. Wynne et al. (2006) confirmed that ascorbic acid did not affect warfarin metabolism at the dietary concentrations (Figure 3).

In our case, the patient had a stable preoperative INR of approximately 1.8, but failed to achieve the same level of anticoagulation after resuming warfarin following bridging therapy. The patient’s dietary intake of vitamin K was consistent with that prior to admission, excluding the possibility of low INR due to high vitamin K consumption. The patient had a history of partial gastrectomy 30 years ago, which could impair gastrointestinal absorption and increase the warfarin dose requirement. However, this was unlikely to cause warfarin resistance, as the patient’s preoperative INR was around 2 with a standard warfarin dose. Moreover, the patient’s genetic test revealed that he had the CYP2C9*3(1075A>C) AA genotype, which conferred warfarin sensitivity and required a lower warfarin dose to achieve the same anticoagulation effect. Despite increasing the warfarin dose to 10mg, the INR remained below the preoperative level. Therefore, other factors might contribute to warfarin resistance in this case. The patient’s medication history suggested that ascorbic acid could be a potential culprit, as the INR returned to the preoperative level after discontinuing ascorbic acid, and the warfarin dose reduction did not affect the INR level, corroborating this hypothesis.

Vitamin C may impair warfarin absorption by affecting the gastric mucosa (Holbrook et al., 2005). Feetam et al. (1975) attributed the decreased absorption of warfarin in subjects who ingested 10 g of ascorbic acid to the diarrheal effects of high doses of ascorbic acid. However, this was not the case for the patient in this report, who did not experience diarrhea during hospitalization. Ascorbic acid could also reduce the anticoagulant effect of warfarin by enhancing warfarin metabolism through enzyme induction, increasing vitamin K activity, and altering the synthesis or degradation of vitamin K-dependent coagulation factors (Wynne et al., 2006). Furthermore, ascorbic acid could act as a chelating agent, which could compromise the efficacy of warfarin (Grebe and Gregory, 2002), but the exact mechanism remains unclear. The variable outcomes of warfarin resistance after ascorbic acid intake in different patients suggested that this drug interaction had considerable individual variability. The mechanism of warfarin resistance induced by ascorbic acid warrants further investigation.

While resistance to warfarin potentially induced by ascorbic acid offers a viable explanation for the observed variations in INR, it is imperative to consider additional contributory elements. These elements encompass dietary patterns that influence vitamin K concentrations, hepatic function alterations affecting drug metabolism, and the simultaneous administration of other pharmaceuticals that may have interactive effects with warfarin. Furthermore, fluctuations in the patient’s overall health, including acute medical conditions or cardiac function shifts, may also bear upon INR values. Adherence to prescribed medication protocols and the precision of INR assessments are additional factors that merit attention. Upon meticulous exclusion of each enumerated factor, we posit that ascorbic acid administration is the most plausible etiology for the warfarin resistance manifested in this case.

## 4 Conclusion

Ascorbic acid-induced warfarin resistance is a rare but potentially serious drug interaction that can compromise the efficacy of anticoagulation therapy. Clinicians should be aware of this possibility and advise patients to avoid taking vitamin C while on warfarin. Patients concomitantly taking vitamin C and warfarin should monitor their INR values closely and discontinue ascorbic acid as soon as possible if they exhibit signs of warfarin resistance.

## Data Availability

The original contributions presented in the study are included in the article/Supplementary Material, further inquiries can be directed to the corresponding author.
